# A preliminary molecular survey of *Babesia divergens* and first evidence of *Theileria annulata* in cattle from Saudi Arabia

**DOI:** 10.14202/vetworld.2019.266-270

**Published:** 2019-02-15

**Authors:** Mohamed W. Ghafar, Sayed A. M. Amer

**Affiliations:** 1 Department of Zoonoses, Faculty of Veterinary Medicine, Cairo University, Egypt; 2 Department of Forensic Biology, College of Forensic Sciences, Naif Arab University for Security Sciences, Saudi Arabia; 3 Department of Zoology, Faculty of Science, Cairo University, Egypt

**Keywords:** *Babesia divergens*, cattle, molecular, Saudi Arabia, *Theileria annulata*

## Abstract

**Background and Aim:**

*Babesia divergens* causes human babesiosis in Europe where the parasite utilizes cattle as animal reservoir and *Ixodes ricinus* as tick vector. Importation of infected animals and passive carriage of infected ticks through migratory birds can lead to tick/pathogen geographic expansion and emergence of diseases in naïve land. Given the information that Saudi Arabia imports cattle from the European countries and that two global bird flyways pass through the country geographic coordinates, we speculate that *B. divergens* might be introduced into the Kingdom. Therefore, the aim of this preliminary study was to molecularly detect and characterize *B. divergens* and other piroplasms (including *Theileria* spp.) in cattle from Taif district, Kingdom of Saudi Arabia.

**Materials and Methods:**

Blood samples from 20 cattle residing Taif district were collected, and polymerase chain reaction tested using wide and species-specific primers. Amplicons from a positive genus-wide reaction were purified, sequenced, and analyzed. Phylogenetic trees were constructed, and similarity to existing GenBank zoonotic piroplasms was also assessed.

**Results:**

All samples were negative for *B. divergens*, and only one sample proved positive for *Theileria annulata* in a wide reaction. Phylogeny clustered our strain with *T. annulata* from Spanish dog and another one detected in a cow from France. BLAST analysis showed genetic distance from zoonotic piroplasms with identity ranged from 88% to 91%.

**Conclusion:**

Although *B. divergens* was not detected, we are not able to rule out or affirm the existence of the pathogen in the country. On the other hand, identifying *T. annulata* strain with a southern European origin strongly supports our speculation that bovine zoonotic Babesia might be introduced into KSA. This study is not only the first molecular survey of *B. divergens* but also the first report of the molecular identity of *T. annulata* in Saudi Arabia. A national-wide bovine and tick surveillance are needed to further prove our speculation.

## Introduction

A large number of piroplasm species belonging to genera *Babesias* and *Theileria* can infect animals, whereas only few Babesias are incriminated to cause human infection. Human babesiosis due to *Babesia divergens* is prevalent in Europe, and the parasite utilizes cattle as animal reservoir and *Ixodes ricinus* as tick vector [1,2].

**Figure-1:**
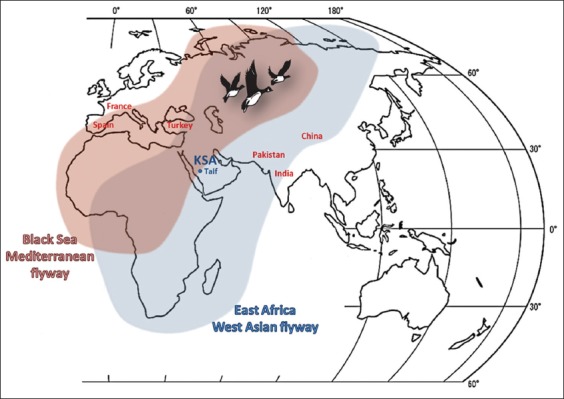
The sampling site, the bird flyways that cross KSA and the countries reported the highest 20 BLAST scoring sequences (Designed by Ghafar MW).

Many possible means can contribute to the tick/pathogen geographic expansion with subsequent emergence of diseases in new areas. One possible controllable mean is the importation of infected domestic or wild animal carrying ticks laden with pathogens [3-5]. Another possible uncontrollable mean is the passive carriage of infected ticks through migratory birds [6,7]. These ways could be applied to *I. ricinus*/*B. divergens* dispersion with the emergence of bovine and/or human babesiosis in naïve locations. Regarding the situation in Saudi Arabia, the Kingdom imports cattle from Europe [8] and two major global bird flyways (the Black Sea/Mediterranean and the East Africa/West Asian) pass through the country geographic coordinates [9] (Figure-1). Given this information, we speculate that *B. divergens* might gain a foothold in Saudi Arabia and posing veterinary and human public health risks.

To the best of our knowledge, there is no work done to demonstrate the existence of zoonotic cattle Babesia in the country. Therefore, the aim of this preliminary study was to molecularly detect and characterize *B. divergens* and other piroplasms (including *Theileria* spp.) in cattle from Taif district, KSA. Detection and characterization of pathogens in new geographic areas help to understand the worldwide epidemiology of the disease and support designing effective prevention and control measures.

## Materials and Methods

### Ethical approval

Blood samples were collected while slaughtering the cattle at Taif abattoir; therefore, no ethical permission was needed.

### Samples and DNA extraction

During 2013, EDTA whole blood samples were collected from 20 apparently healthy cattle during slaughtering at Taif abattoir. These animals were residing at Taif district (approximately 21° 26’ 14” N and 40° 30’ 45” E), KSA (Figure-1). Samples were labeled and sent under refrigeration to Biotechnology Laboratory at Taif University.

Total genomic DNA purification was performed using AxyPrep Blood Genomic DNA Miniprep Kit (Cat. No. AP-MN-BL-GDNA-250) according to the manufacturer protocol. Extracted DNA was stored at −20^o^C till used in polymerase chain reaction (PCR).

### PCR and sequencing

Amplifications were performed in 25-µl reaction mixtures containing 5 µl of each DNA template, 12.5 µl GoTaq Green Master Mix (Promega Corporation, Madison, WI 53711-5399, USA), and 20 pmoles of each primer. All samples were tested using *Babesia*-F (GTG-AAA-CTG-CGA-ATG-GCT-CA) and *Babesia*-R (CCA-TGC-TGA-AGT-ATT-CAA-GAC) primer pair. This oligonucleotide set targets the common sequence of the 18S rRNA gene of the genus *Babesia* [10]. The following thermocycle profile was used: initial 5-min denaturation at 95°C, 34 cycles (each consisting of 30-s denaturation at 95°C, 30-s annealing at 55°C, and a 1.5-min extension at 72°C) and a 5-min final extension at 72°C. Products of ~650 bp indicate positive results. All specimens were also subjected to PCR testing using *B. divergens*-specific primers (5’-GTT-TCT-GAC-CCA-TCA-GCT-TGA-C-3’ and 5’-CAA-TAT-TAA-CAC-CAC-GCA-AAA-ATT-C-3’) [11]. This primer pair also targets 18S rRNA gene and for which the following thermocycle profile was used: an initial 5-min denaturation at 95°C, 34 cycles (each consisting of a 30-s denaturation at 95°C, a 30-s annealing at 61°C, and a 45-s extension at 72°C) and a 5-min final extension at 72°C. Amplicons of ~353 bp indicate positive results. Amplified products were analyzed on 1.25% agarose gel by electrophoresis and seen under UV with ethidium bromide. Amplicons were purified from agarose gel using FavorPrep Gel Purification Mini Kit (Cat. No. FAGPK001) as directed by the manufacturer. Extracted products were subjected to bidirectional sequencing using Macrogen facilities.

### Sequence analysis and phylogeny

The sequenced 18S rRNA gene fragment (502 bp) in the present study was analyzed by BLAST (https://blast.ncbi.nlm.nih.gov/Blast.cgi). Two manually aligned files were generated using MacClade v.4 [12] to construct the most parsimonious phylogenetic trees. The first file contained our sequence with several selected numbers of designated *Babesia* and *Theileria* species for the identification of the organism to the species level based on similarities. To give detailed molecular and epidemiological information, a second file included our strain with the highest 20 scoring GenBank sequences was generated to expand the positioned cluster. Ambiguous and gap-containing sites were deleted so that 386-bp and 427-bp were left in the aligned files for the first and second phylogenetic analysis, respectively. The phylogenetic tree was constructed using the neighbor-joining method [13] using PAUP*4.0b10 [14]. The tree method was adjusted with tree bisection-reconstruction as branch-swapping algorithm and 10,000 bootstrap replications with heuristic searches. The 18S rRNA gene sequences of *Plasmodium falciparum* (accession no. M19172) and *Theileria ovis* (Accession no. MG333458) were used to root the trees. Additional BLAST analysis was performed to figure out similarity of the 18S rRNA of our strain with existing GenBank human pathogenic piroplasms. Our partial 18S rRNA nucleotide sequence (*Theileria annulata -* Ghafar) has been deposited in GenBank (Accession no. LC379205).

## Results

**Figure-2:**
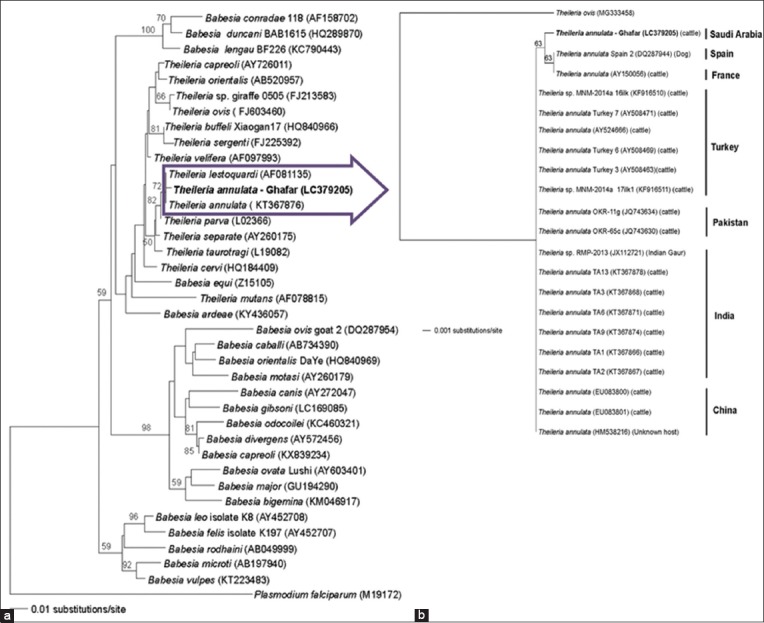
Neighbor-joining trees of 18S rRNA gene sequences showing relationships of our strain with previously designated *Babesia* and *Theileria* species (a) and with top 20 scoring BLAST sequences (b).

All samples proved negative when tested using standard PCR with *B. divergens*-specific primers, while only one (1/20, 5%) sample showed amplification in a genus-wide reaction. Based on BLAST analysis and GenBank sequence database, the molecular identification of our partial 18S rRNA gene sequence showed that the detected piroplasm belonged to *T. annulata* (100% query coverage, 99% sequence identity, and 0 E-value). The phylogenetic analysis using selected sequences (Figure-2a) placed our sample on a separate cluster with *T. annulata* and *T. lestoquardi*. The other phylogenetic tree constructed to expand the proposed cluster using top 20 scoring *T. annulata* GenBank sequences (Figure-2b) revealed that our strain is positioned on a branch with *T. annulata* isolated from a dog from Spain and another strain detected in a cow from France. The other 18 *T. annulata* sequences (six from Turkey, two from Pakistan, seven from India, and three from China) were located on the same branch. Table-1 demonstrates the similarity features of the 18S rRNA of our *T. annulata* Ghafar strain with existing GenBank human pathogenic piroplasms using BLAST. Our strain proved genetically distant from zoonotic species with identity ranged from 88% to 91%.

**Table-1:** Similarity features of our strain with existing GenBank human pathogenic piroplasms using BLAST (Accessed May 22, 2018).

Organism	Isolate/strain	Maximum score	Total score	Query cover (%)	E-value	Identity (%)	Accession#
*Babesia microti*	GI	689	689	100	0.0	91	AF231348
*Babesia microti*	TC-2012-C1	689	689	100	0.0	91	KF410827
*Babesia microti*	Gray	689	689	100	0.0	91	AY693840
*Babesia microti*	Jena/Germany	689	689	100	0.0	91	EF413181
*Babesia microti*	TC-2012-B1	684	684	100	0.0	91	KF410824
*Babesia microti*	449-L	682	682	100	0.0	91	JQ609304
*Babesia microti*	Kobe524	682	682	100	0.0	91	AB032434
*Babesia microti*	TC-2012-B87	680	680	99	0.0	91	KF410825
*Babesia microti*	TC-2012-B99	673	673	99	0.0	91	KF410826
*Babesia divergens*	Rouen 87	647	647	100	0.0	90	FJ944822
*Babesia divergens*	CF2000	647	647	100	0.0	90	FJ944823.
*Babesia* spp. Human KY	Human KY	647	647	100	0.0	90	AY887131
*Babesia* spp. MO1	MO1	647	647	100	0.0	90	AY048113
*Babesia* spp. BAB693W	BAB693W	641	641	100	0.0	90	AY274114
*Babesia divergens*	-----	641	641	100	0.0	90	AJ439713
*Babesia divergens*	B_di09	641	641	100	0.0	90	EF458228
*Piroplasmida* gen. spp. WA1	WA1	640	640	100	0.0	89	AF158700
*Piroplasmida* gen. spp. WA2	WA2	640	640	100	0.0	89	AF158701
*Babesia duncani*	BAB1615	640	640	100	0.0	89	HQ289870
*Babesia duncani*	BAB2	640	640	100	0.0	89	HQ285838
*Babesia venatorum*	-----	636	636	100	0.0	90	KM244044
*Babesia venatorum*	BAB20	636	636	100	0.0	90	AY046575
*Babesia* spp. WA1	CA5	634	634	100	0.0	89	AY027815
*Babesia* spp. WA1	CA6	634	634	100	0.0	89	AY027816
*Piroplasmida* gen. spp. CA3	CA3	616	616	100	8e-179	89	AF158704
*Piroplasmida* gen. spp. CA4	CA4	616	616	100	8e-179	89	AF158705
*Piroplasmida* gen. spp. CA1	CA1	616	616	100	8e-179	89	AF158703
*Babesia* spp. KO1	KO1	586	586	100	6e-170	88	DQ346955
*Theileria*-related spp.	-----	566	566	90	8e-164	89	L13730

## Discussion

Several plausible explanations could account for the negative detection of *B. divergens*. The first, most unlikely, explanation is that the whole country including Taif district is free of the disease as the pathogen never entered the area. The second, unlikely, explanation is that the disease is introduced into the country, but due to the absence of competent vector, the pathogen could not be perpetuated. The third, least unlikely, explanation is that the disease arrived at the land somewhere rather than Taif district and remained localized due to lack of suitable environmental conditions for the carried ticks. The fourth, likely, explanation is that the pathogen arrived into Saudi Arabia including Taif district; however, using of small-sized sample (20 cows) led to the production of biased results. Taking into account the above-mentioned exposition, it is difficult to address conclusions to exclude or affirm the presence of *B. divergence* in the country. To accurately determine existence of the disease in KSA, a national survey testing large-sized cattle sample collected from different localities should be performed. Additional steps of testing different tick species collected from the environment and while attached to cattle and migratory birds are needed to prove or rule out our speculation thoroughly.

Using genus-wide primers made it possible to identify a piroplasm of genus *Theileria*. Detection of *T. annulata* in this study was not a surprise as the parasite is endemic in the area and several studies reporting its occurrence in Saudi cattle were published [8,15,16]. However, this study is considered the first report documenting the molecular identity of the pathogen in KSA.

Phylogenetic analysis with the highest 20 scoring *T. annulata* GenBank sequences clustered our sample with a canine strain from Spain and another one detected in a cow from France. This result substantially promotes our speculation that pathogens could be introduced from Europe into KSA. There are some facts that robustly support our result including: (1) Southern and Eastern Spain as well as Southern France constitute parts of Mediterranean Europe, a region that shares climate, flora, and fauna [17], (2) the Black Sea/Mediterranean bird flyway passes through the Mediterranean Europe regions [9], (3) *T. annulata* was detected in a Spanish dog and the sequence data analysis revealed that it is identical to that of *T. annulata* from cows residing in Southern Spain [18], and (4) sequence information of *T. annulata* isolates from France was identical to that from Spain [18]. Given the previously mentioned result and facts, we can conclude that our Ghafar strain has a southern European origin. Apart from national cattle trade and bird migration, importation of dogs from Spain would account as another way for the introduction of *T. annulata* into KSA.

The other highest 18 scoring *T. annulata* GenBank sequences (six from Turkey, two from Pakistan, seven from India, and three from China) were located on the same tree branch. This result also correlates well with the pattern of East Africa/West Asian bird flyway, supporting the role of migratory birds in dissemination of tick-transmitted pathogens.

Our *T. annulata* Ghafar strain showed genetic distance from human pathogenic piroplasms with identity ranged from 88% to 91%. This result correlates hitherto with the reality that *T. annulata* has not been reported to infect humans before.

## Conclusion

Although *B. divergens* could not be detected in cattle samples in the current study, we are not able to rule out or affirm the existence of the pathogen in the country. On the other hand, identifying a *T. annulata* strain, which has a southern European origin, strongly supports our speculation that bovine zoonotic babesia may be gained a foothold in KSA. This study is not only the first molecular survey looking for *B. divergens* but also the first documentary report of the molecular identity of *T. annulata* in Saudi Arabia. A national-wide survey testing a large number of cattle samples collected from different areas is needed to address the presence of *B. divergens* in bovine host. Furthermore, additional testing of different tick species collected from the environment and while attached to cattle and migratory birds are needed to further prove our speculation.

## Authors’ Contributions

MWG designed the study, collected the samples and materials, and performed the experiments. SAMA conducted molecular and phylogenetic analyses. Both authors prepared the figures and wrote the manuscript. All authors read and approved the final manuscript.
